# Case Report: A case report of the diagnosis of renal inflammatory myofibroblastic tumor

**DOI:** 10.3389/fonc.2025.1543858

**Published:** 2025-06-06

**Authors:** Wenjie Wu, Tiantian Liang, Hui Zhang

**Affiliations:** ^1^ Department of Nuclear Medicine Department, Shanxi Traditional Chinese Medical Hospital, Taiyuan, China; ^2^ Department of Radiology, Guizhou Provincial People’s Hospital, Guiyang, China; ^3^ Department of Radiology, First Hospital of Shanxi Medical University, Taiyuan, China

**Keywords:** inflammatory myofibroblastic tumors, kidney, computed tomography, magnetic resonance imaging, pathologic diagnosis

## Abstract

Renal inflammatory myofibroblastic tumor is a very rare disease that primarily occurs in the renal parenchyma and may even involve the renal pelvis. This study presents a case of renal inflammatory myofibroblastic tumor with exophytic growth, whose imaging appearance resembles that of a solitary perirenal mass. A 75-year-old male patient was referred to our hospital for further treatment after a retroperitoneal lesion was discovered and suspected at another hospital. Imaging examination revealed multiple cysts in both kidneys and a lesion located anterior to a cyst at the lower pole of the left kidney. Due to the presence of the cysts, the relationship between the lesion and the kidneys was unclear. We performed multiplanar reconstruction of the tumor images and generated a temporal signal curve. Based on these imaging findings, we determined that the lesion was a benign tumor of renal origin and could be surgically removed. Finally, pathological examination confirmed the diagnosis of a renal inflammatory myofibroblastic tumor. Postoperative telephone follow-up revealed that the patient was alive, and a follow-up abdominal CT performed at the local hospital showed no recurrence.

## Introduction

Inflammatory myofibroblastic tumors (IMT) are distinct fibroblastic and myofibroblastic tumors of intermediate biological potential, characterized by marked inflammatory infiltrates, primarily composed of lymphocytes and plasma cells (1). In the genitourinary tract, the urinary bladder is the most common site of IMT, while renal origin is rare (2). According to previous case reports, renal IMTs can arise in the renal parenchyma, renal pelvis, or both, and rarely exhibit exophytic growth. Renal IMT can be clinically silent, present with flank pain, or cause microscopic and macroscopic hematuria (3, 4). We present a case of renal IMT with exophytic growth, whose imaging appearance resembles that of a solitary perirenal mass.

## Case presentation

A 75-year-old male patient presented to a local hospital with general malaise and a low-grade fever. Upon admission, CT examination of the whole abdomen revealed multiple cysts in both kidneys and a left retroperitoneal mass. Following anti-inflammatory treatment and symptomatic treatment, the patient’s condition improved, and he was discharged. Two months later, the patient was admitted to our hospital for further evaluation and treatment. Since the onset of the disease, the patient’s spirit, sleep, and appetite have been good; urination and defecation have been normal, and there has been no significant weight loss. The patient has a history of bilateral basal ganglia lacunar infarction but denies any history of hypertension, heart disease, diabetes, hepatitis, or tuberculosis. He has undergone bilateral inguinal hernia repair. Physical examination revealed no obvious abnormalities, and routine laboratory tests, including hematuria screening, were unremarkable.

**Figure 1:**
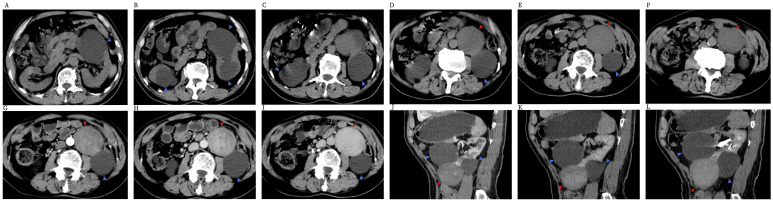
**(A–F)** Abdominal unenhanced scan CT. Blue arrows indicate cysts; red arrows indicate masses. Multiple cysts are visible in both kidneys. A solid, uniform mass is observed anterior to a cyst in the lower pole of the left kidney, with a CT value of approximately 37 HU. The lesion has a regular morphology and well-defined margins. **(G–I)** Abdominal contrast-enhanced CT images. **(G)** Mild heterogeneous enhancement of the lesion is seen in the arterial phase. **(H)** In the portal phase, the lesion shows progressive enhancement (CT value: 93 HU). **(I)** In the delay phase, the lesion shows gradual enhancement (CT value: 95 HU). **(J–L)** Sagittal CT images: **(J)** arterial phase, **(K)** portal phase, **(L)** delay phase. The lesion is located between two giant cysts in the left kidney and is connected to its lower pole. The contrast-enhanced scan shows mild, gradual enhancement of the lesion.

Abdominal CT was performed after admission, revealing multiple cysts in both kidneys, with the largest located at the middle of the lateral margin of the left kidney. A solid, homogeneous mass measuring approximately 6.0 cm × 6.5 cm was observed anterior to the cyst at the lower pole of the left kidney, exhibiting a CT value of about 37 HU. The lesion had a regular morphology and clear margins (**Figures 1A–F**). Enhanced scanning showed mild, gradual enhancement of the lesion (CT values of 57 HU in the arterial phase, 93 HU in the portal phase, and 95 HU in the delayed phase), indicating uptake of iodinated contrast and suggesting the presence of blood supply within the mass (**Figures 1G–I**). However, the degree of enhancement remained lower than that of normal renal parenchyma. The perirenal fat space was clear, with no thickening of the perirenal fascia and no significant enlargement of retroperitoneal lymph nodes. There were no signs of invasion into adjacent organs or metastatic disease in the abdomen.

The relationship between the lesion and the kidney was unclear due to the influence of cysts. We performed multiplane reconstruction of CT images to obtain sagittal and coronal images, which better demonstrated the relationship between the lesion and surrounding tissues. From the sagittal images (**Figures 1J–L**), we determined that the lesion was located between two giant cysts in the left kidney and was connected to the lower pole of the left kidney. While we assessed the lesion’s origin and its relationship with surrounding organs through CT, the findings provided limited valuable information about the lesion itself. Therefore, we recommended abdominal MRI to further characterize the lesion.

**Figure 2:**
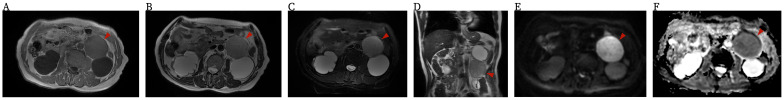
MRI plain scan: **(A)** T1WI, **(B)** T2WI, **(C)** T2WI with fat saturation, **(D)** T2WI in the coronal position, **(E)** DWI (*b* = 800 s/mm²), and **(F)** ADC map. Red arrows indicate mass. A mass is visible in front of the cyst at the lower pole of the left kidney. The lesion shows equal signal on both T1WI and T2WI, high signal intensity on DWI, and slightly low signal on the ADC map, with well-defined boundaries and limited diffusion.

**Figure 3:**
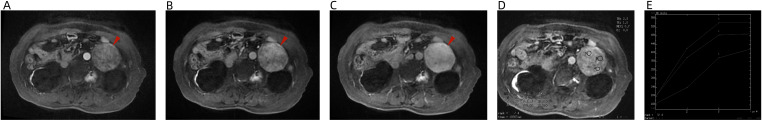
MRI enhancement: **(A)** arterial phase, **(B)** portal phase, **(C)** delay phase, **(D)** ROI, and **(E)** TIC. The lesion shows mild, gradual enhancement. The TIC demonstrates a wash-in pattern, indicating a gradual inflow of contrast agent into the lesion tissue, which suggests that the lesion may be a benign tumor.

An MRI plain scan revealed a mass anterior to the cyst at the lower pole of the left kidney. The lesion exhibited isointense signals on both T1- and T2-weighted images, high signal intensity on DWI sequence scans, and slightly low signal on ADC maps, with clear lesion boundaries and limited internal diffusion (**Figure 2**). Enhanced scanning demonstrated gradual enhancement of the lesion (**Figures 3A–C**). A region of interest (ROI) was selected on the lesion to generate a time-signal intensity curve (TIC). The curve (**Figures 3D, E**) showed a wash-in pattern, reflecting the gradual inflow of contrast agent into the lesion tissue, suggesting that the lesion may be benign.

In a multidisciplinary meeting, we agreed that the lesion was a benign tumor based on imaging findings. Due to the lesion’s particular location, the patient exhibited no obvious clinical symptoms. Considering the lesion’s clear boundary, regular shape, absence of obvious enlargement of retroperitoneal lymph nodes, no signs of invasion of nearby organs, and no evidence of abdominal metastasis, we unanimously agreed that the lesion could be locally removed by surgery.

The patient underwent retroperitoneal laparoscopic decompression of the left renal cyst and resection of the retroperitoneal mass. A longitudinal incision was made in the perirenal fascia, extending from the diaphragmatic apex down to the level of the upper edge of the iliac fossa. The perirenal fat sac was opened to expose the surface of the left kidney, revealing a cystic mass approximately 4.0 cm × 5.0 cm located at the middle of the lateral edge of the left kidney, without adhesion to the surrounding tissues. The cyst wall was punctured, and the cyst fluid was clear. After aspirating the cyst fluid, the cyst wall was circumferentially resected approximately 0.5 cm from the edge of the renal parenchyma using an ultrasonic scalpel. It was found that the cyst did not communicate with the left kidney collection system, and no obvious abnormality was observed at the base. The cyst wall was sent for frozen pathology examination, which confirmed it was a simple cyst. Dissection continued toward the lower pole of the left kidney, where a tumor of approximately 8.0 cm × 7.0 cm was observed, clearly related to the left kidney. The mass was separated and exposed using an ultrasonic scalpel. After the fat on the surface of the tumor and the lower pole of the left kidney was freed and cleaned, the connection between the tumor and the kidney was clamped and completely resected. The resected tissue was sent for pathological examination.

**Figure 4:**
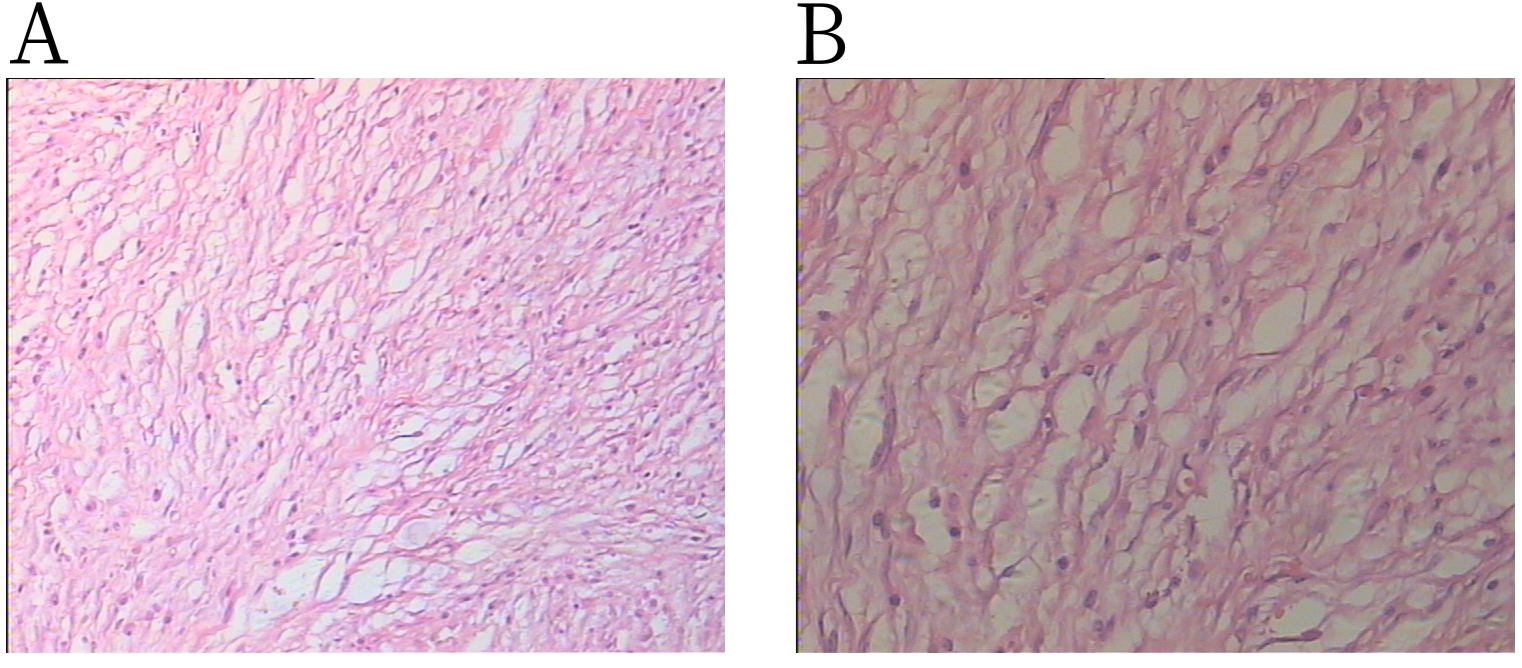
H&E staining shows spindle cells admixed with lymphoplasmacytic infiltration: **(A)** × 100 magnification and **(B)** × 200 magnification.

Pathology revealed that the tumor was mainly composed of spindle cells and short spindle cells with inflammatory cell infiltration (**Figure 4**). The pathological diagnosis was an inflammatory myofibroblastic tumor. Immunohistochemistry results were as follows: Anaplastic Lymphoma Kinase (ALK) (+), CD117 (−), CD34 (+), CD38 (+), desmin (−), Dog-1 (−), HMB 45 (−), ki-67 (+), s-100 (−), Smooth Muscle Actin (SMA) (−), vimentin (+), and CD138 (+).

Postoperative recovery proceeded without complications. Following thorough deliberations at a multidisciplinary meeting, the patient was discharged on the sixth postoperative day. At telephone follow-up 7 years later, the patient was still alive. As the patient had undergone follow-up examinations at a local hospital, we were unable to obtain the imaging data.

## Discussion

IMT is a rare mesenchymal tumor of intermediate biological potential, characterized by spindle cell proliferation accompanied by inflammatory cell infiltration (1). IMT occurs in various parts of the genitourinary system, most commonly in the bladder and less frequently in the kidney (2). Based on previous case reports of renal inflammatory myofibroblastic tumors, renal IMT can arise in the renal parenchyma, renal pelvis, or both; it may be clinically silent, present with flank pain, or manifest as microscopic and macroscopic hematuria (3, 4). Renal IMT was first reported in 1972 (5), and there is no significant difference between sex and age at which renal IMT occurs. The causes of IMT remain unclear. According to the literature, chronic inflammation, surgery, and trauma are considered predisposing factors for IMT (6). Currently, the WHO classifies this lesion as a fibroblastic and myofibroblastic tumor of intermediate malignancy (rarely metastasizing) (7).

Renal IMT usually presents with lumbar spine or abdominal pain, microscopic hematuria, or gross hematuria, and some patients seek treatment due to unexpected findings during physical examination. According to previous case reports, IMT is typically treated with nephrectomy or partial resection, with a few being treated with corticosteroids; however, there is currently no uniform, accurate, and effective treatment standard for IMT (6). The prognosis of renal IMT surgery is relatively good. Kapusta et al. (8) reported 12 cases of renal IMT; eight cases were followed up for 1 to 17 years, and no postoperative recurrence was observed.

The imaging manifestations of kidney IMT are nonspecific and inconclusive, making it difficult to distinguish renal IMT from other neoplastic lesions in the kidney by imaging examination. On CT imaging, most kidney IMTs appear as isodense or slightly hypodense masses, with cystic degeneration, necrosis, calcification, or invasive growth. After contrast enhancement, the masses show uniform or heterogeneous enhancement to varying degrees. Kidney IMT often exhibits variable signal intensity on MRI T1-weighted imaging (T1WI) and low signal intensity on T2WI (6). In our case, the lesion exhibited uniform, slightly lower density and clear boundaries. The enhanced scan showed mild, gradual enhancement; this enhancement method may be related to the gradual filling of contrast agent into the collagen fiber space or increased vascular permeability caused by inflammatory lesions, although the degree of enhancement remains lower than that of normal renal parenchyma. Therefore, it is difficult to distinguish the lesion from other kidney tumors based on imaging alone. However, imaging can determine the lesion’s size, location, relationship with adjacent tissue, and renal involvement. Ultimately, the diagnosis of kidney IMT still depends on pathological examination.

In our case, the patient’s clinical symptoms were not obvious, and the location of the lesion differed from those reported in previous case studies. According to earlier reports, renal inflammatory myofibroblastoma is more commonly found in the renal parenchyma and may even involve the renal pelvis, but it rarely grows outward. Liang (9) reported a case report of renal inflammatory myofibroblastic tumor resembling cystic renal cell carcinoma, in which the entire cystic-solid lesion protruded beyond the contour of the kidney. In this case, the lesion exhibited an exophytic growth pattern. Due to the presence of multiple huge cysts in the left kidney, the lesion appeared similar to an independent mass outside the kidney, which led to uncertainty about its origin. On the CT sagittal image, the lesion was clearly seen to be sandwiched between two large cysts in the left kidney, and the size of the cysts was close to that of the lesion. Therefore, we suspected that the huge cysts might have caused the lesion to grow outward. Regarding the nature of the lesions—benign or malignant—we agreed that it was likely benign based on its imaging characteristics and TIC curve. We considered the possibilities of renal angiomyolipoma (spent fat) or renal leiomyoma. We had not considered a renal inflammatory myofibroblastic tumor, as this disease is exceedingly rare.

Diagnosis of renal inflammatory myofibroblastic tumor relies on pathological diagnosis. The main pathological component of renal inflammatory myofibroblastoma is spindle cells, with additional components including variable extracellular matrix collagen and chronic inflammatory cells (plasma cells, lymphocytes) (7). Immunohistochemical analysis showed that the tumor cells of inflammatory myofibroblastoma strongly expressed vimentin, partially expressed desmin and ALK, and showed no expression of CD34 and CD117. Some studies have also confirmed the myofibroblastic characteristics of IMT, with expression of SMA, myosin, vimentin, and CD34 (10). In this patient, immunohistochemistry showed ALK (+) and vimentin (+), consistent with previous reports.

## Conclusion

Renal inflammatory myofibroblastic tumor is a rare, low-grade malignant tumor that is difficult to distinguish from other renal tumors preoperatively based on clinical symptoms, laboratory examinations, and imaging findings. It is mainly confirmed by postoperative pathological and histological staining, but CT and MRI can still play an important role in judging the invasion range of surrounding tissues and blood vessels, providing an important reference for the clinical treatment plan. They can also be applied to efficacy evaluation after IMT treatment and early postoperative detection of tumor recurrence.

## Data Availability

The original contributions presented in the study are included in the article/Supplementary Material. Further inquiries can be directed to the corresponding author.
